# Prioritizing Investment in Medical Education

**DOI:** 10.1371/journal.pmed.0030159

**Published:** 2006-03-28

**Authors:** Fawad Aslam

**Affiliations:** **1**Lahore Cantt, LahorePakistan

The dire need to reform medical education in South Asia has been well emphasized in the *PLoS Medicine* Editorial [ 1]. It is encouraging to note that efforts are under way to devise strategies to bring about this reformation. However, for such reforms to be effective, it is crucial that the opinions of medical students and young doctors are also taken into account. Students' roles should be enhanced from those of mere consumers of medical education to those of contributors [ 2]. They are important stakeholders, and their active participation in policymaking will facilitate the creation of more robust solutions.

The need for drastic improvement in health research in South Asia is well established. The need for research in medical education is perhaps even greater. Unfortunately, indigenous data pertaining to medical education in this region are limited. Only a small number of studies have attempted to explore the concerns of students and doctors in matters pertaining to, for example, medical decision making and health research [ 3, 4]. The establishment of a research culture is fraught with difficulties but is not impossible [ 5]. It is my opinion that, to bring about reform, both a “bottom-up” and a “top-down” approach are needed. The former needs ample student exposure to research during medical school. The latter is essentially linked to the availability of funds. No amount of community-oriented training, for example, will compensate for the deficiency of properly qualified health professionals in rural areas. It is only when there is sufficient financial and professional security that the greater purpose of educational reform will stand fulfilled. It is hard to envisage how this can be achieved when the bulk of budgetary spending pertains to debt-servicing and defense expenditure.

Alongside medical education, parallel investment should be sought in health education, not only because our physicians are not cognizant of current treatment practices [ 6], but also because our patients have a poor knowledge of common diseases that afflict them [ 7]. The interaction of better-informed patients and properly qualified doctors may significantly improve community health. For impoverished nations, the importance of preventive medicine is manifold as it offers the most economical way of combating disease. There is some evidence to suggest that our medical students are not “prevention” oriented, and, thus, more emphasis must be placed on preventive medicine [ 8].

It is also hoped that such investment will lead to nationally oriented research activities and not to a mere replication of Western studies. The study evaluating the significant protective effects of hand washing in children from common childhood diseases is one such example [ 9]. Another example is a study evaluating the effects of garlic on dyslipidemia [ 10]. Further studies of this kind may prove helpful in combating the cardiovascular disease epidemic in Pakistan. Garlic is potentially a much cheaper alternative to statins, the latter being unaffordable for most segments of Pakistani society. Similarly, medical education institutions such as Aga Khan University in Pakistan, which is a private-sector entity, have started problem-based, community-oriented teaching in medical schools. The outcome of these curricular changes remains to be seen. Indeed, there is hope for South Asia, but for such hope to materialize, we need selfless individuals, strong institutions, and perhaps above all a more just and realistic distribution of the national financial resources.
